# Supplementary material for: Effectiveness of Home-Based Cardiac Rehabilitation, Using Wearable Sensors, as a Multicomponent, Cutting-Edge Intervention: A Systematic Review and Meta-Analysis
Source: J Clin Med. 2022 Jun 29;11(13):3772. doi: 10.3390/jcm11133772 (PMC9267864; doi:10.3390/jcm11133772)
Supplement: Supplementary file 1 [file jcm-11-03772-s001.zip › Supplementary Figures.pdf]

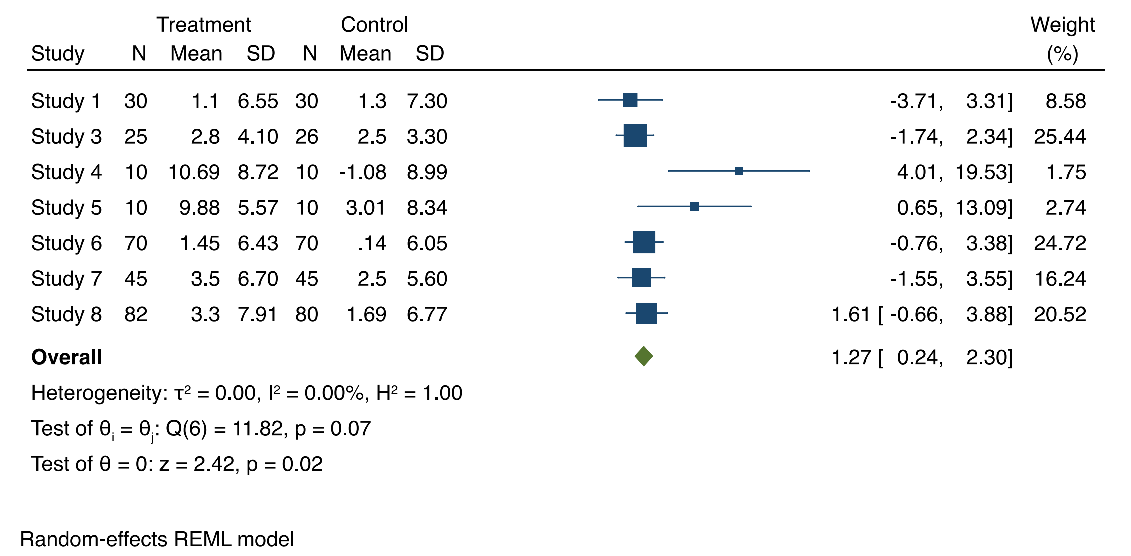

Supplementary Figure S1. Results from the Restricted Maximum Likelihood (REML) random effects meta-analysis concerning the mean difference in cardiorespiratory fitness (CRF)(ml/kg/min) change post-intervention, between the home-based cardiac rehabilitation group(HBCR) and the center-based cardiac rehabilitation(CBCR).

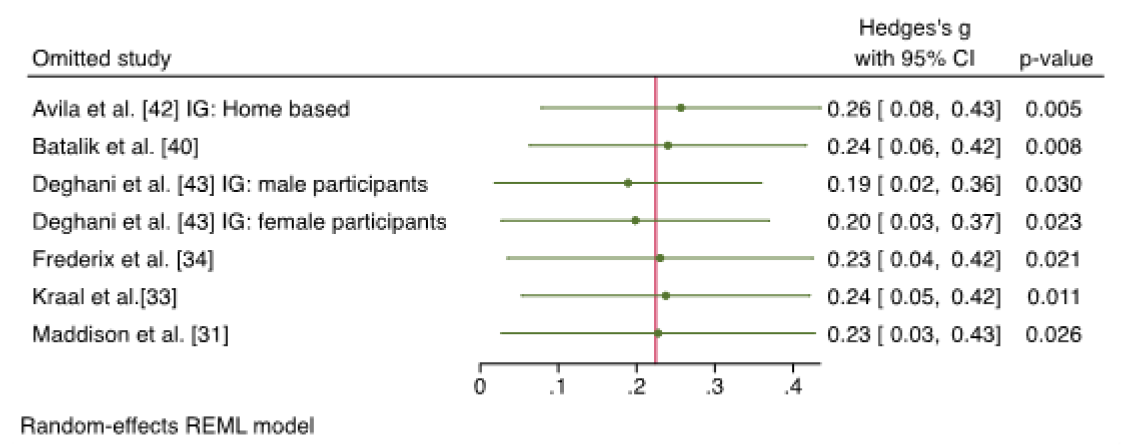

Supplementary Figure S2. Results from the Leave-one out meta-analysis between the home-based cardiac rehabilitation group (HBCR) and center-based cardiac rehabilitation (CBCR).

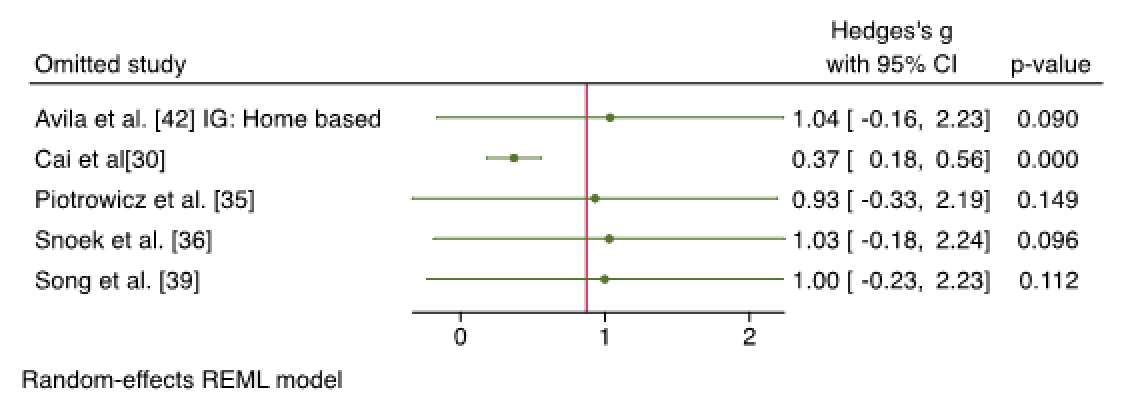

Supplementary Figure S3. Results from the Leave-one out meta-analysis between the home-based cardiac rehabilitation group (HBCR) and the usual care group (UC).

Heterogeneity analysis

```
fect-size label: Hedges's g
        Effect size: _meta_es
          Std. err.: _meta_se
        Study label: Study

Meta-analysis summary                                    Number of studies =      5
Random-effects model                                     Heterogeneity:
Method: REML                                                          tau2 =  1.1945
                                                                    I2 (%) =   96.41
                                                                        H2 =   27.85

-----------------------------------------------------------------------------------
                           Study |     Hedges's g    [95% conf. interval]  % weight
---------------------------------+-------------------------------------------------
Avila et al. [42] IG: Home based |          0.221      -0.280       0.722     19.77
                  Cai et al[30] |          2.894       2.335       3.452     19.52
         Piotrowicz et al. [35] |          0.653       0.243       1.063     20.11
              Snoek et al. [36] |          0.269      -0.024       0.562     20.46
               Song et al. [39] |          0.382      -0.019       0.783     20.14
---------------------------------+-------------------------------------------------
                           theta |          0.872      -0.106       1.850
-----------------------------------------------------------------------------------
Test of theta = 0: z = 1.75                                     Prob > |z| = 0.0806
Test of homogeneity: Q = chi2(4) = 72.94                          Prob > Q = 0.0000
```

Supplementary Figure S4. Heterogeneity analysis of the included studies comparing the home-based cardiac rehabilitation group (HBCR) versus the usual care group (UC).
